# An Updated View on the Molecular Pathomechanisms of Human Dihydrolipoamide Dehydrogenase Deficiency in Light of Novel Crystallographic Evidence

**DOI:** 10.1007/s11064-019-02766-9

**Published:** 2019-03-07

**Authors:** Attila Ambrus

**Affiliations:** Department of Medical Biochemistry, MTA-SE Laboratory for Neurobiochemistry, Semmelweis University, 37-47 Tuzolto Street, Budapest, 1094 Hungary

**Keywords:** Dihydrolipoamide dehydrogenase, E3 deficiency, Alpha-ketoglutarate dehydrogenase complex, Pyruvate dehydrogenase complex, Pathogenic mutation, Reactive oxygen species (ROS), X-ray crystallography, Structure

## Abstract

Dihydrolipoamide dehydrogenase (LADH, E3) deficiency is a rare (autosomal, recessive) genetic disorder generally presenting with an onset in the neonatal age and early death; the highest carrier rate has been found among Ashkenazi Jews. Acute clinical episodes usually involve severe metabolic decompensation and lactate acidosis that result in neurological, cardiological, and/or hepatological manifestations. Clinical severity is due to the fact that LADH is a common E3 subunit to the alpha-ketoglutarate, pyruvate, alpha-ketoadipate, and branched-chain alpha-keto acid dehydrogenase complexes, and is also a constituent in the glycine cleavage system, thus a loss in LADH function adversely affects multiple key metabolic routes. However, the severe clinical pictures frequently still do not parallel the LADH activity loss, which implies the involvement of auxiliary biochemical mechanisms; enhanced reactive oxygen species generation as well as affinity loss for multienzyme complexes proved to be key auxiliary exacerbating pathomechanisms. This review provides an overview and an up-to-date molecular insight into the pathomechanisms of this disease in light of the structural conclusions drawn from the first crystal structure of a disease-causing hE3 variant determined recently in our laboratory.

## E3 Deficiency—the Disease, Enzyme, and Affected Multienzyme Complex Functions

(Dihydro)lipoamide dehydrogenase (LADH, E3; gene: *dld*) deficiency is an often prematurely lethal rare autosomal recessive genetic disorder [1]; the highest carrier rate (1:94-1:110, G194C-hE3, h for human) has been found among Ashkenazi Jews with a disease frequency of 1:35,000–1:48,000 [2, 3]. The first and sole review on the molecular pathomechanisms of this disease was written by our laboratory [4]. This disorder involves mainly neurological, cardiological, and hepatological manifestations whose symptoms generally arise very early in life. The phenotypic spectrum includes hyperammonemia, failure to thrive, hypotonia, encephalopathy, seizure, hepatomegaly, liver dysfunction, lactate acidosis, hypoglycemia, Leigh syndrome, developmental delay, hypertrophic cardiomyopathy, vision impairment/optic atrophy, ataxia, and microcephaly, among others [1, 3, 5–16]. Potentially lethal hepatological consequences, often together with encephalopathy and coagulopathy, may present in isolation and in adulthood [3, 17–19]. The severity of the clinical outcomes is due to the simultaneous defects of the mitochondrial E3-harboring multienzyme dehydrogenase complexes for alpha-ketoglutarate (KGDHc), pyruvate (PDHc), alpha-ketoadipate (KADHc), and branched-chain alpha-keto acids (BCKDHc); interestingly, the glycine cleavage system (GCS), which also contains the LADH protein, remains unaffected in E3-deficiency [1, 4, 20–22]. In the above dehydrogenase complexes the common E3 subunit catalyzes the re-oxidation of the dihydrolipoate (DHLA) moieties covalently linked to the respective E2 components and the reduction of NAD^+^ to NADH (LADH activity, forward reaction). Pathogenic gene variants include missense or nonsense mutations, splice site variants, and ones with (small) deletions/insertions; 14 disease-causing enzyme variants have been reported to date in the clinical literature [4, 6, 8].

## Auxiliary Exacerbating Pathomechanisms

Clinical severity, more often than not, does not parallel the loss in LADH activity [4, 6, 8, 13, 20, 23, 24]. Recent results suggest that the missing clues could be (i) enhanced reactive oxygen species (ROS) production by various pathogenic hE3 mutants [20, 23, 24], particularly in acidosis [24], (ii) liberation of selected hE3 variants from the E3-tethering multienzyme complexes [25–29], and (iii) ROS generation by the E1–E2 subcomplex of the hKGDHc (when E3 is scarce) [20], principally in the course of acidosis [30].

### ROS Generation by hE3, Pathogenic hE3 Mutants, and hE3-Harboring Multienzyme Complexes

**Scheme 1:**
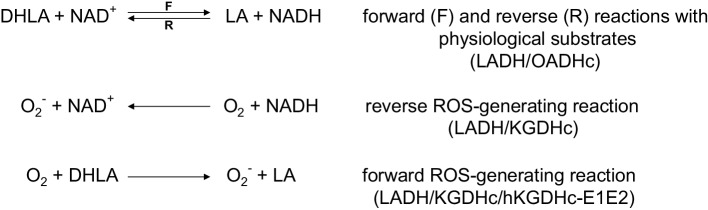
Forward, reverse, and ROS-generating reactions of LADH

Among all the mitochondrial alpha-keto (or 2-oxo) acid dehydrogenase complexes (OADHc), the KGDHc exhibits the most dominant ROS generation under pathologically relevant conditions [20–22, 31–42]. ROS generation by the KGDHc can occur in the forward catalytic direction in case the physiological electron acceptor NAD^+^ is scarce or absent (in vitro), or alternatively in the reverse reaction driven by a high NADH/NAD^+^ ratio [20, 30, 33, 34]; superoxide (as a primary ROS) is generated at the flavoenzyme E3 component [20, 33, 34] (see Scheme 1). The isolated E3 component [43, 44] is also capable of generating ROS, in an oxidase reaction, in either direction of the catalytic reaction [20, 24, 30, 45–51] (Scheme 1). The E3 subunit forms a functional (obligate), non-covalent homodimer that uses residues from both monomers for the physiological LADH activity [45, 52–55], but not for the ROS-generating activity [56–58]; hE3 comprises four domains: a FAD-binding (1–149), a NAD^+^/NADH-binding (150–282), a central (283–350), and an interface domain (351–474). ROS generation in the reverse reaction of the isolated E3 component is stimulated in acidosis; this same pathological condition also enhances the ROS production in the reverse, but not in the forward reaction of KGDHc [30]. Sensitivity to a decreasing pH of ROS-generation by isolated disease-causing hE3 mutants that display increased ROS-generating capacities in the reverse reaction was reported to be even more pronounced [24]; pathogenic substitutions which stimulated ROS production took place at the disulfide-exchange site (P453L), the dimerization surface (E340K, D444V), or the cofactor-binding site (G194C) [24]. Calibrated gel filtration, molecular dynamics (MD) simulation, hydrogen–deuterium exchange mass spectrometry (HDX-MS), diffusion-ordered (DOSY) NMR, (soft-ionizing) nano-LC MS, and X-ray crystallography (see below) all confirmed that neither (mild) acidosis nor the hitherto investigated disease-causing homodimerization surface mutations led to monomerization of the E3 dimer [24, 25, 30, 59–62]. Importantly, ROS production could also be stimulated by a relevant disease-causing hE3 mutant (G194C-hE3) when complexed to a multienzyme complex (hKGDHc) [20]. The D444V-, G194C-, E340K-, R460G-, and R447G-hE3 pathogenic mutants were reported to oxidatively deteriorate the lipoic acid (LA) cofactors of the PDHc and KGDHc in a yeast model, and in case of D444V-hE3, in human homozygous fibroblasts [23]. In mutants exhibiting stimulated ROS production, LADH activity was generally impaired in both catalytic directions. In P453L-hE3 the physiological activity was almost entirely lost while the ROS-generating activity became predominant, whereas in G194C-hE3 the LADH activity was not altered, but the ROS-producing capacity increased [24]. For P453L-hE3 the clinical phenotype was very severe [16, 63] and the excessive ROS production was proposed to be a contributing factor to this [24]. G194C-hE3 leads often to adult-onset manifestations, which is in accord with the retained LADH activity and the moderately enhanced ROS generation [24]. FAD contents were almost entirely retained in D444V-hE3 and E340K-hE3, while G194C-hE3 and P453L-hE3 exhibited ~ 30% loss of FAD [24]. Circular dichroism (CD) spectroscopy represented no significant overall structural alterations in the above four mutants [24]. HDX-MS however detected significant changes in flexibility/exposure in the lipoic acid (LA) binding channel of P453L-hE3 and G194C-hE3, which could potentially result in stimulation of ROS production. HDX-MS data for D444V-hE3 and E340K-hE3 were rather inconclusive in terms of the mechanism of action of increased ROS production [25]. MD simulation of 13 disease-causing hE3 mutants was also carried out [59, 60]; good correlation with HDX-MS results was reported for the P453L, K37E, G194C, I445M, and R460G substitutions [4].

#### Conclusions of the First Disease-Causing Mutant Structure (D444V-hE3) Relevant to Compromised Enzymatic Activity and ROS Generation

**Fig. 1:**
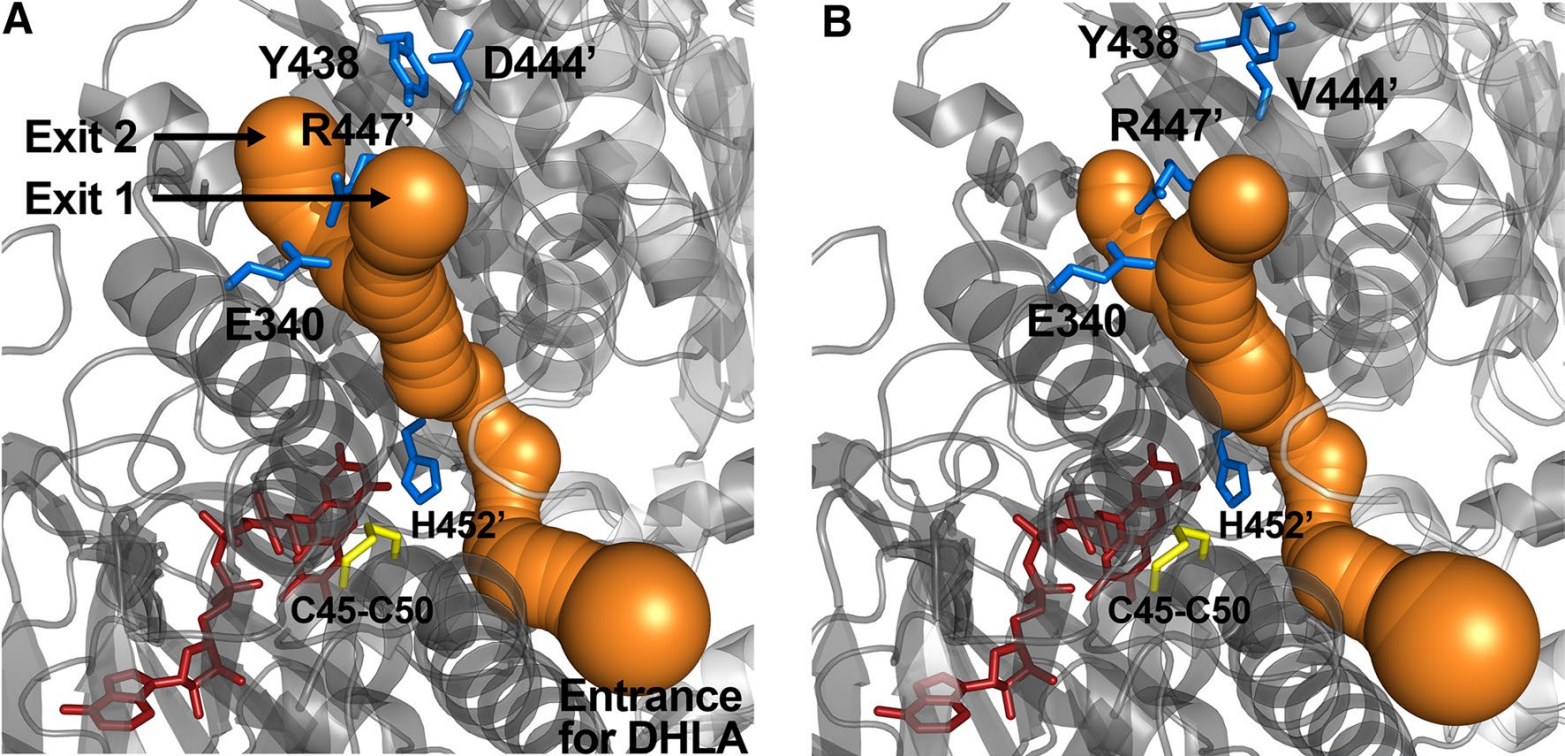
The LA(/DHLA)-binding and H^+^/H_2_O channels in the A-B dimers of hE3 (A, PDB ID: 5NHG) and D444V-hE3 (B, PDB ID: 5J5Z). Monomer A is labeled in both proteins. The redox-active C45-C50 pair and FAD are represented as yellow and red sticks, respectively. (Color figure online)

**Fig. 2:**
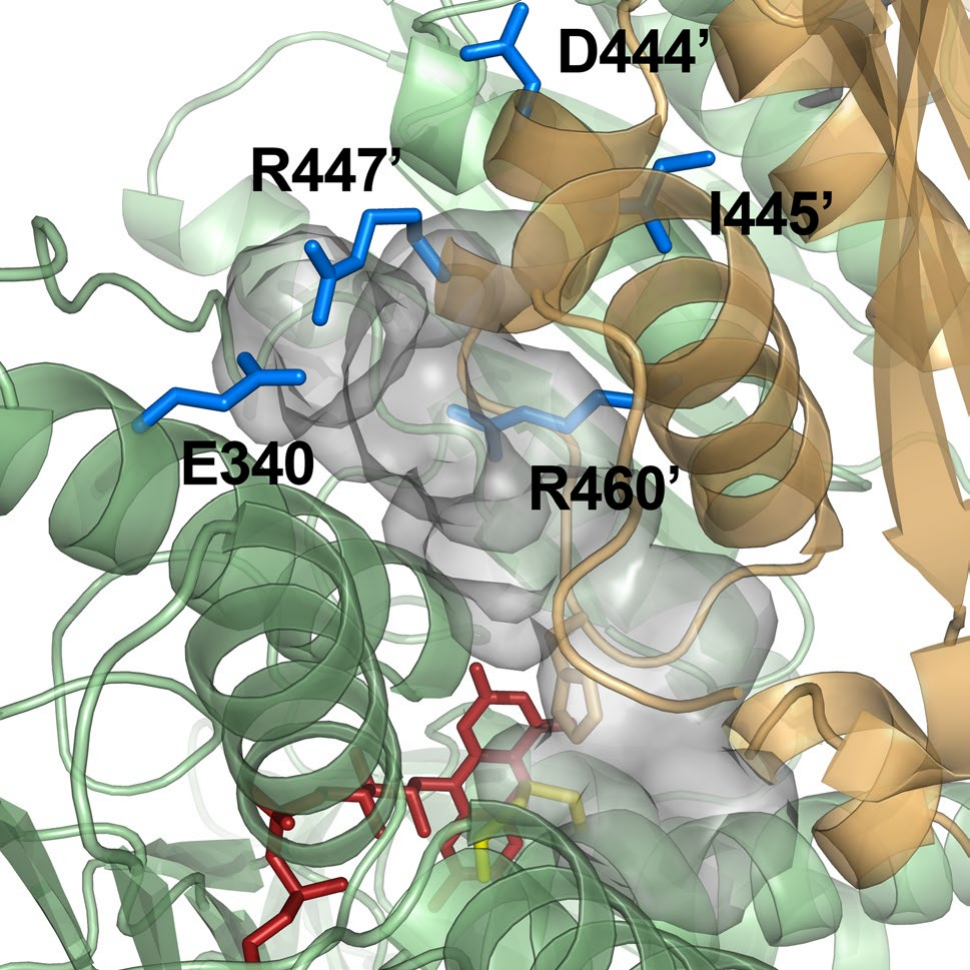
Pathogenic amino acid substitution sites near the H^+^/H_2_O channel in the hE3 crystal structure (PDB ID: 5NHG). The inner surface of the channel is displayed (A-B dimer). The C45-C50 pair and FAD are colored as for Fig. 1. (Color figure online)

Crystal structures have been published for hE3 [28, 29, 52, 61, 64] and now also for the D444V-hE3 disease-causing mutant from our laboratory [61]. High-resolution crystal structures have very recently been determined in our laboratory also for the P453L- (PDB ID: 6I4Z), G194C- (PDB ID: 6I4P), R460G- (PDB IDs: 6I4R and 6HG8), R447G- (PDB ID: 6I4S), I445M- (PDB ID: 6I4T), and G426E-hE3 (PDB ID: 6I4U) disease-causing variants and for hE3 at the hitherto highest 1.75 Å resolution (PDB ID: 6I4Q), however, the thorough and comparative analysis of these structures is still in progress. The already published and analyzed D444V-hE3 crystal structure demonstrated a shorter and wider H^+^/H_2_O-releasing channel when compared to the wild type structure. This channel is solvent accessible, leads to the active site and it is the continuation of the LA-binding substrate channel (Fig. 1); the H^+^/H_2_O channel appears to have catalytic roles in LADH function and perhaps ROS generation by hE3 [61]. A drop in surface potential around the exit of this channel was also found when compared to the hE3 structure. Several helices and random coils form the above channel, the helices all pointing with the positive ends of their dipoles towards the active site; structural alterations in the channel-forming helices likely affect the active site *via* relayed helix dipole moment contributions. All the above mentioned structural alterations in D444V-hE3 hence indeed may lead to a drop in enzyme activity and the positive shift in ROS-generating capacity [61]. Another indirect effect that might also have contribution to the pathological behaviors is an overall change in penetration through the channel upon structural changes, which was suggested altering the apparent redox potential of the FAD moiety [65, 66]; the redox status of FAD has direct influence on both the regular catalytic and ROS-generating activities. The C-terminus in D444V-hE3 was shown by HDX-MS to possess higher flexibility as compared to hE3 [25]; this effect could not be detected by crystallography, perhaps due to the cryogenic conditions. Since the C-terminus separates and hence forms connection between the LA-binding and the H^+^/H_2_O channels, any change in this region might also affect the LA-binding substrate channel, which is also implicated in both the normal catalytic action as well as superoxide generation [25, 61]. Since the disease-causing dimer interface substitutions (D444V, E340K, R447G, R460G, I445M) all take place in the vicinity of the H^+^/H_2_O channel (Fig. 2), considerably far from the LA-binding channel, the cofactor-binding sites, and the active site, this channel was connected to the potential existence of a generalized pathomechanism of human E3-deficiency for the disease-causing dimerization interface mutations [61].

### Affinity Loss for Multienzyme Complexes and Respective Structural Conclusions for D444V-hE3

The affinity of E3 for the KGDHc proved to be low [67–69] and even lower in acidosis [30]; E3 binds ~ 30 times stronger to the PDHc [68, 70, 71]. Several experimental evidence suggest that E1, and not E2, would directly bind E3 in the KGDHc [72–74]. LADH can also exist as a liberated protein in vivo [30, 68, 75–78]; it is the most abundant flavoprotein in brain and muscle mitochondria [79]. Several disease-causing hE3 variants (R447G-, D444V-, R460G-, and E340K-hE3) exhibit significantly impaired affinity for the hPDHc leading to greatly compromised overall hPDHc activities [25–27, 29]. The D444V-hE3 crystal structure demonstrated a drop in surface potential over the entire protein molecule, while HDX-MS showed an enhanced flexibility on the surface where the E3-binding protein (E3BP) of hPDHc is tethered [25, 61]; both effects likely contribute to the compromised affinity for hPDHc. The D444V-hE3 crystal structure also confirmed previous experimental data on the lack of monomerization and FAD loss in this mutant [61].

### ROS Generation by the E1–E2 Subcomplex of the hKGDHc

In case hE3 is untethered from the hKGDHc, as is likely the case for several pathogenic variants and in acidosis, the E1-E2 subcomplex is potentially also capable of generating ROS at a very considerable rate in the forward catalytic direction [20] (Scheme 1). Thus, under such conditions, ROS production might proceed simultaneously from E3 (principally in the reverse catalytic direction) as well as from the E1-E2 subcomplex (in the forward catalytic direction), provided that substrate provision is sufficient [4, 20]; an intact population of KGDHc may still retain some overall activity [6], unless the LA prosthetic group suffers oxidative damage [23]. Since the E2 (dihydrolipoamide succinyltransferase) component could potentially also be targeted against ROS generation, we very recently determined its cryo-electronmicroscopic structure; we were able to detect residues 218–453 at 3.31 Å resolution (PDB ID: 6H05), which in a further and still progressing stage of structure determination appears to develop to be 2.9 Å resolution (unpublished result).

## Conclusions

In conclusion, human E3-deficiency is still an incurable and hardly manageable disease, which might be taken under better control by dietary restrictions, nutritional support, correction of metabolic acidosis and coagulopathy, administration of flavins, lipoic acid, thiamine, trial of dichloroacetate, for selected and respective cases [1, 4]. Since in many cases with impaired affinity for multienzyme complexes the residual hE3 activity is much higher than the residual overall multienzyme complex activity, with the high-resolution crystal structures in hand, adaptor molecules could potentially be developed to retether the respective pathogenic hE3 mutants to their harboring multienzyme complexes; although this might only be a limited therapeutic solution, the approach can potentially be advantageous for selected patients. Development of specific ROS generation inhibitors against selected hE3 mutants and the E2 subunit of the hKGDHc, besides a general antioxidant therapy, might also be a valid approach towards possible therapeutic solutions. Besides gene therapy, which still requires substantial development to be efficient and completely safe, enzyme replacement therapy might gain potentials in the treatment of human E3 deficiency in the near future [80, 81].
